# Differences Between Pediatric Extra-Pulmonary and Pulmonary Tuberculosis: a Warning Sign for the Future

**DOI:** 10.4084/MJHID.2014.058

**Published:** 2014-09-01

**Authors:** İlker Devrim, Hüseyin Aktürk, Nuri Bayram, Hurşit Apa, Şener Tulumoğlu, Fatma Devrim, Tülin Erdem, Gamze Gulfidan, Yüce Ayhan, İpek Tamsel, Demet Can, Hüdaver Alper

**Affiliations:** 1 Department of Pediatric Infectious Diseases, Dr. Behçet Uz Children’s Hospital, İzmir, Turkey; 2 Department of Clinical Microbiology, Dr. Behçet Uz Children’s Hospital, İzmir, Turkey; 3 Pediatric Allergy and Asthma Unit, Dr. Behçet Uz Children’s Hospital, İzmir, Turkey; 4 Department of Pediatrics, Dr. Behçet Uz Children’s Hospital, İzmir, Turkey; 5 Department of Radiology, Ege University, Faculty of Medicine, İzmir, Turkey

## Abstract

**Background:**

Tuberculosis (TB) remains a major global health problem. The childhood tuberculosis has some unique features different which makes the diagnosis more complicated. Here we described the epidemiologic, clinical and microbiologic features of children with extra pulmonary and pulmonary TB.

**Methods:**

The data of the patients <14 years with active TB were collected and compared in pulmonary (PTB) and extrapulmonary TB (EXPTB) patients.

**Results:**

A total of 128 cases was included. Forty-two cases occurred in children were < 5 years of age; 41 cases between 6–10 years and 45 cases > 10 years. PTB was present in 75,0% of the cases, and EXPTB was present in 25% of cases. There was no significant difference between the EXPTB and PTB by means of distribution of age groups (p=0,201). The rate of patients free of constitutional symptoms were significantly higher in EXPTB compared to PTB(p=0,000). There was no significant difference between EXPTB and PTB by means of sources detection(p=0,069).

**Conclusion:**

TB is still a major public health problem. EXPTB has an insidious and silent onset without any constitutional symptoms, and both microbiological confirmation and the source by an adult are not frequently found. Moreover, detection of the adult source is mandatory for controlling the TB disease in children

## Introduction

Tuberculosis (TB) remains a major global health problem affecting millions of people annually. It is the second leading infectious cause of death following human immunodeficiency virus (HIV) worldwide.^1^ In 2011, 9 million new cases and 1.4 million deaths due to TB were reported. Despite difficulties for estimation of TB, 500000 new cases and 64000-deaths among children under 15 years old were expected in 2011.^1^ Recent data from Turkey; estimated the incidence rate of TB as 24 (21–27) / 100000 population including HIV-POSITIVE population, being the incidence of HIV-TB population only 0.04 (0.04–0.05) / 100000.^2^

TB is still a major health problem for children as well as adults. Although children constitute 5% of TB population in low-burden countries, it is reported to be as high as 20–40% in other countries.^3^–^6^ Turkey has an additional risk for outbreaks of TB comparing to Europe and US; because of large numbers immigrants from war-zones of Middle East such as Syria had been travelling across the country.

Tuberculosis in children has unique features, different from adults, which make the diagnosis more difficult. The symptoms of TB in children have a broad spectrum changing from non-specific symptoms to severe clinical presentations. Although pulmonary involvement is frequent, all organs can be involved.^7^,^8^ Almost every child in Turkey has Bacillus Calmette–Guérin (BCG) vaccination at least once, thus the tuberculin skin test (TST) has limited benefit at diagnosis. Although previous studies suggested whole-blood interferon-gamma release assays (IGRAs) for confirmation of exposure in TST positive children, discrimination of latent and active TB disease could not be done based on this test as the value of the test have not been well studied in young children and infants;^9^,^11^ moreover IGRAs are not routinely performed in clinical settings in Turkey. Thus, the diagnosis and decision to start therapy are based on clinical suspicion supported by history of exposure, clinical features, chest radiograph and TST as well as some degree of IGRAs.^12^,^13^

In this study, we reviewed our experience at pediatric patients with active TB admitted in a tertiary hospital in a 5-year period in Turkey. We aimed to compare the epidemic, clinical and microbiological features of the extra-pulmonary tuberculosis (EPTB) and pulmonary tuberculosis (PTB) in children.

## Methods

Patients under 14 years of age diagnosed with active TB in our unit between December 2008 and September 2013 were included. Medical records of children suspected of having tuberculosis hospitalized in Dr. Behcet Uz Children’s Hospital (Izmir, Turkey) were reviewed. Data including demographic characteristics, clinical history, microbiology, imaging studies, medications and outcomes of the patients were collected from medical records. The microbiologically confirmed active TB was defined as the presence of at least one positive clinical specimen for *Mycobacterium tuberculosis* (Mtb) in culture, or positive AFB smear microscopy, or histological confirmation of caseating granulomas.^14^ Probable tuberculosis was defined as the presence of suggestive radiologic signs in the absence of bacteriologic confirmation, or radiologic certainty, positive nucleic acid amplification test for Mtb and good clinical response to antitubercular treatment.^15^

Tests including TST, radiologic studies, conventional x-ray graphs, computerized tomography (CT), cranial magnetic resonance imaging (MRI); microbiological and molecular tests were used for the diagnosis of TB. Purified protein derivate of five units was used for TST.

### Sample processing

2454 samples from 1024 patients were available for analysis, including sputum (n=511), gastric fluid (n=1633), urine (n=61), cerebral spinal fluid (CSF) (n=14), bronchoalveolar lavage fluid (n=47) and other sterile body fluids (n=188). All of the samples, except sterile body fluids, were processed by standard decontamination using Mycoprosafe (NaOH 2%-Trisodium Citrate %1,47, Salubris, Turkey).

Kinyoun acid fast-stain smear examination, Lowenstein Jensen cultivation and automatized culture were performed to all samples. As a result of the establishment of molecular analysis system at recent year (2013) PCR was performed in a limited number of gastric fluid (n=282), sputum (n=87), urine (n=50), 27 bronchoalveolar lavage fluid (n=27), CSF (n=4) and other sterile body fluids (n=54) samples from 504 patients

Smear, cultures and PCR tests were performed as described previously in accordance with standard protocols and/or manufacturers’ protocols:

### Kinyoun acid fast-stain smear

Two drops (approximately 200 μl) of processed sample deposit were placed on a slide and stained by Kinyoun fast staining method according to the conventional methodology

### Lowenstein Jensen cultivation

Two drops of each deposit was inoculated into a Lowenstein Jensen tube media (GBL, Turkey) tube following the manufacturer’s protocol. The culture was incubated in a CO_2_ incubator at 37°C for 42 days.

### Bac/Alert 3D system

Five hundred microliters of each deposit was inoculated into a supplemented Bact/Alert MP bottle following the manufacturer’s protocol (Biomerieux, France). The cultures were incubated in Bact/Alert 3D automatized system at 37°C, and the results automatically reported.

### Identification of Mycobacteria

Five hundred microliters of each culture (Bact/Alert) positive samples was inoculated into a supplemented Bact/Alert MP bottle in which 200 microliters 4-nitro benzoic acid (Aldrich, U.S.A.) solution prepared by following the manufacturer’s protocol was added. For controls, a second sample was inoculated into a 4-nitro benzoic acid free Bact/Alert MP bottle. The cultures were incubated in Bact/Alert 3D automatized system at 37°C, and the results automatically reported. The samples that show no cultivation in 4-nitro benzoic acid containing medium but positive in the control bottle were identified as Mycobacterium tuberculosis complex. The samples with duplicate cultivation were identified as atypical Mycobacteria.

### PCR

The PCR system (Expert MTB/RIF, Cepheid, U.S.A) integrates and automates sample processing, nucleic acid amplification, and detection of the target sequences in simple or complex samples using real-time PCR and reverse transcriptase PCR. The PCR test was conducted simultaneously with conventional procedures on decontaminated and sterile samples following the manufacturer’s standard operating procedure. One and a half milliliters of sample reagent was added to 0.5 ml of processed sample, and incubated at room temperature for 15 minutes with intermittent shaking and finally added to the test cartridge and loaded onto the machine. The result was available after 2 hours.

### Rifampin resistance

For any sample with a positive result for rifampicin resistance by Expert MTB/RIF, the corresponding isolate from the same sample isolated by Lowenstein Jensen or Bact/Alert culture was tested for RIF susceptibility in an external mycobacterium reference laboratory. The primers in the Xpert MTB/RIF assay amplify a portion of the *rpoB* gene containing the 81 base pair “core” region.

Tests for HIV antibodies were performed with commercial kits (Du Pont, Wilmington, DE). Patients were grouped into two groups including EXPTB and PTB patients for further analysis. White blood cells (WBC), C-reactive protein (CRP) and erythrocyte sedimentation rate (ESR) were compared between EXPTB and PTB groups. CRP levels were classified into three groups as a < 2, 2–10 and 10–20 mg/l.

This study was approved by the Local Research Ethics Board of Dr. Behçet Uz Children’s Hospital.

### Statistical analysis

SPSS, version 13.0 (SPSS Inc, Chicago, USA) was used for statistical analyses. Independent samples t-test or a non-parametric analog, Mann-Whitney U test, were used to compare the means of two independent groups. The Chi-square or Fisher’s exact test were used to test for equality of proportions between groups. *P* < 0.05 was considered statistically significant.

## Results

### Demographic data

A total of 129 cases of active TB was identified. Among them, 12 cases having both EPTB and PTB manifestations were excluded from the study. The mean age of the TB patients was 7.0±4.5 years (ranging from 3 months to 14 years of age). Forty-three subjects (36.8%) were under 5 years of age, and 74 (63.2%) were older than ten years of age. In our study; 15 (12.8%) of the patients were under one year of age.

### Clinical presentation

Ninety-two (78.6%) of the cases had PTB and 25 (21.4%) of the cases had EPTB (Table 1). Although six cases were diagnosed as miliary TB according to the CT images, they were then regrouped as PTB since no extrapulmonary focus was found. Sixty of the patients were female (51.3%), and 57 patients (48.7 %) were male. There was no significant difference between EXPTB and PTB groups according to the gender and age (p=0.94; p=0.31).

Table 2 summarizes the main clinical and microbiological characteristics of the children with PTB, EXPTB and the whole active TB group. The most common signs and symptoms on admission were fevers in 40 cases (34.2%) and cough in 81 cases (69.2%). The incidence of cough was significantly higher in the PTB group (81.5%) when compared with EXPTB group (36.0%) (p<0.05). The number of patients without symptoms including fever, cough, malaise and weight loss were significantly higher in EXPTB (72.0%) group when compared with patients in PTB group (13.0%) (p<0.05).

Only 37.1% of the total patients had higher leucocyte counts while 53.0% of children had increased CRP values. ESR was in normal range in 47.0% of the patients. There was no significant difference between children with PTB and EXPTB by means of WBC, CRP and ESR (p> 0.05) (Table 3)

TST was done in all subjects and was found to be positive in 74 (63.2%) of the patients. We found no significant difference between PTB and EXPTB groups by means of TST positivity (p=0.075). In this study; TST was anergic in 11 cases (9.4%) including two patients with miliary TB, eight with PTB and one with TB peritonitis. Only 15 patients had IGRA’s thus this data were not included in further analysis.

The source of infection was determined in 44 of the patients (37.6%), including father (n=21), mother (n=5), grandparents (n=7), siblings (n=3), secondary relatives and neighbors (n=8). In eight cases; there was more than one person as a source of infection in households. The detection rate of source in PTB group (42.4%) was significantly higher than the rate in EPTB group (20.0%) ( p=0.04). In the drug-resistant group, no source of infection could be established in 5 of 9 patients (55.5%). There was no significant difference between patients younger than 5 years of age and 5 to 14 years of age by means of detection of the source (p=0.26).

### Microbiology

The diagnosis of active TB was microbiologically confirmed by Ziehl–Neelsen staining, culture or PCR. Ziehl–Neelsen staining was positive in 14 cases (11.9%) of 117 patients. PCR was performed in 86 patients with positive results in 17 cases (22.9%). The culture positivity in samples of sputum or gastric lavage was found in 15 of 117 cases (12.8%).

Drug susceptibility test results for isoniazid (INH), rifampicin (RIF), ethambutol (EMB), and pyrazinamide (PYZ) were available in 15 isolates. Resistance to INH was present in five patients (33.3 %). Resistance to both INH and RIF (defined as multi-drug resistance, MDR) was detected in 2 (6.6 %) cases. Two cases were resistant to all of INH, RF, EMB and PYZ. As further analysis for other anti-Tb drugs was not available in our institution, two patients were referred to another health care center for this purpose.

### Treatment

TB was successfully treated in 115 patients (98.2%) with clinical and microbiological improvement. Two patients were found to be resistant to the standard first-line anti-TB drugs. We had two relapses in one-year follow-up time (0.85%).

## Discussion

Every organ could be the target organ of TB. The diagnosis of EPTB is a difficult challenge and frequently delayed since the symptoms are non-specific depending on the affected sites. In our study, the number of patients without symptoms including fever, cough, malaise and weight loss were significantly higher in EPTB and 5 times more than PTB, in accordance with previous findings().^8^,^16^ Moreover, the incidence of cough was significantly higher in the PTB group (81.5%) compared to EPTB group (36.0%) (p<0.05). Absence of classic signs and symptoms of cough, fever, night sweats, weight loss, anorexia or fatigue in EPTB is the main factor for delays in diagnosis. Therefore, invasive procedures are requested for an early and certain diagnosis; of course, invasive procedures are more troublesome and dangerous in children. In our opinion; the typical findings and constitutional symptoms are not necessarily present especially in EPTB and diagnosis require high clinical suspicion.

In our study the majority of the TB cases (78.6%) were associated with pulmonary involvement supporting the findings of the recent papers from Italy, Denmark and Turkey.^11^,^14^,^17^,^18^ Nearly 2/3 of the patients had EPTB alone suggesting that EPTB could be underdiagnosed in the absence of pulmonary findings. Lymphadenopathy (LAP) was the most prominent finding in isolated EPTB. CNS involvement was found in one patient in our study. Current articles have reported LAP as the common site of extra pulmonary involvement^14^ however some reports had suggested that CNS involvement in TB could be as high as 15.8%.^14^,^18^ A recent study from China reported the incidence of TB meningitis as 38.8% in TB.^19^ The low incidence of CNS TB in our study would be due to the high rate of BCG vaccination. The protective features of BCG for meningeal disease, despite the unequal efficiency between different geographic regions, would be a possible mechanism for this finding.^20^

Age could be an important factor in TB development and progression. Children have a higher probability of progression comparing to the adults. Possibility of developing severe and extra pulmonary forms of TB was reported to be more frequent in children under five years of age.^11^ A recent study from Italy suggested higher TB rates in children under 5 years of age,^14^ however in our study the majority of the cases were found to be older than 5 years, suggesting that the distribution of age could show differences from country to country.

Finding the source of TB infection is essential for controlling the TB burden. In our study; 37.6% of the patients; had no detectable source of infection. Moreover, the source of TB infection could not be determined in 80% of the EPTB patients while this ratio decreases to 57,6% in PTB patients. The rate of detection of the source in children was reported in 28.0% and 38.8%;^14^,^19^ moreover in a recent study a negative history of contact was associated with extra-pulmonary localizations, supporting our findings.^14^ In our study, negative contact history did not show any statistical difference between age groups, this could be because mostly adult contacts were parents or grandparents in our country, and the presence at day-care center before 5 years of age is not frequent. The negative contact history was present nearly half of the drug-resistant TB cases; which indirectly reflects undetected adult patients infected with resistant mTB. This conclusion is in accordance with the recent report in which only 1.8% of children had developed TB as household contacts with adult TB patients.^21^

Acute-phase reactants were reported to be high in patients with TB although they are not specific to TB.^22^ Among these reactants, CRP has been suggested as a candidate biomarker for active infection with M. tuberculosis.^22^,^23^ Moreover, Kumat et al. reported that CRP, besides other molecules, could be used for distinguishing PTB from EPTB.^23^ On the contrary to this article; neither CRP nor ESR was found to be high in PTB compared to EPTB in our study.

The World Health Organization (WHO) estimates that HIV prevalence among children with TB was changing from 10 to 60%^24^ depending on the prevalence of HIV. In our clinic, only one patient who was diagnosed as HIV infection had suffered from TB demonstrating its low incidence in our study. In developed countries such as England, two children with HIV per year were presented with active TB over 5 year period with a total of 5.5% of HIV infected patients, had TB^25^ supporting our findings.

In our study, INH resistance was found to be high as 33.0%; however, this high proportion was thought to be due to the low number of isolated mycobacteria. In a recent systemic review of 95 studies including 8,351 children with TB, the median proportion of children INH resistant was 8%.^26^ In one study from Turkey; frequency of INH-resistant TB was reported as 6.7 % in children.^27^ However, this various findings supported that isoniazid-resistant tuberculosis in children is a widespread and geographically variable phenomenon.

## Conclusion

EPTB diagnosis is more difficult than PTB in children due to the various problems such as absence of associated pulmonary involvement, lack of constitutional symptoms and negative TB exposure history compared to PTB. Even acute phase reactants failed to be useful in discrimination of EPTB. New strategies are required for improving the diagnosis of EPTB in children

## Figures and Tables

**Table 1:** Organ involvement of TB in children

Organ Involvement	N (%)

Pulmonary TB	92(78.6)

Miliary TB	6 (5.1)

Extra-pulmonary TB	19 (16.2)

Skin	1(0.8)
Lymph node	13(11.1)
Pericardium	2(1.7)
Peritoneum	2(1.7)
Meningitis	1(0.8)

**Table 2:** Clinical symptoms of the patients with pulmonary TB and extrapulmonary TB.

	Pulmonary TB	Extra-pulmonary TB	Total	P value
Clinical symptoms				
Cough	75( 81.5%)	6(24.0%)	81(69.2%)	<0.05
Fever	31( 33.7%)	9(36.0%)	40( 34.2%)	0.829
Malaise	18(19,6 %)	4 (16.0%)	22(18.8.%)	0.781
Weight loss	24(26.1%)	5(20.0%)	29 (24.8%)	0.532
Night sweats	26( 28.3%)	4(16.0%)	30(25.6%)	0.303
No fever and cough-malaise-weight loss* (constitutional symptoms)	12(13.0%)	18(72.0%)	30(25.6%)	<0.05
TB contact history	39(42.4%)	5(20.0%)	44(37.6%)	<0.05
TST positivity	74 (63.2%)	62(67.4%)	12(48.0%)	0.075

* All the symptoms were absent at the same time

**Table 3:** Laboratory of the patients with pulmonary TB and extrapulmonary TB.

	Pulmonary TB	Extra-pulmonary TB	Total	P value
Increased White blood cell count	35( 381.5%)	8(18.6%)	43(37.1%)	0.67
ESR				
<20	42 (45.7%)	13(52.0%)	55(47.0%)	0.818
20–60	31 (33.7%)	8 (32.0%)	39(33.3%)	
>60	19 (20.7%)	4 (16.0 59	23(19.7%)	
CRP				
<2	57 (62.0%)	17( 68.0%)	74(63.2%)	0.601
2–10	26( 28.3%)	6 (24.0%)	32(27.4%)	
>10	9 (9.8%)	2(8.0%)	11 (9.4%)	
